# Characterization and phylogenetic analysis of the mitochondrial genome of *Macdunnoughia hybrida* (Lepidoptera: Noctuidae: Plusiinae)

**DOI:** 10.1080/23802359.2021.1945972

**Published:** 2021-07-14

**Authors:** Yuanchen Zhang, Shuang Xue

**Affiliations:** College of Biology and Food Engineering, Anyang Institute of Technology, Anyang, Henan, China

**Keywords:** Noctuidae, mitochondrial genome, *Macdunnoughia hybrida*, phylogenetic analysis

## Abstract

*Macdunnoughia hybrida* is a polyphagous herbivorous moth within the family Noctuidae. In this study, we sequenced and analyzed the complete mitochondrial genome (mitogenome) of *M. hybrida*. This mitogenome was 15,382 bp long and encoded 13 protein-coding genes (PCGs), 22 transfer RNA genes (tRNAs), and two ribosomal RNA unit genes (rRNAs). Except for *cox1* started with CGA, all other PCGs started with the standard ATN codons. Most of the PCGs terminated with the stop codon TAA, whereas *cox1*, *cox2* and *nad4* end with the incomplete codon T−−. The whole mitogenome exhibited heavy AT nucleotide bias (80.7%). Gene order was conserved and identical to most other previously sequenced Noctuidae. Phylogenetic analysis positioned *M. hybrida* in a well-supported clade with *Diachrysia nadeja* and three *Ctenoplusia* species. The relationships (Catocalinae + (Plusiinae + (Acronictinae + (Heliothinae + (Amphipyrinae + (Hadeninae + Noctuinae)))))) were supported within Noctuidae.

The Noctuidae, commonly known as owlet moths, cutworms or armyworms, are the most controversial family in the superfamily Noctuoidea because its classification is still contingent (Regier et al. 2017). As the second largest family in Noctuoidea, it has 1089 genera and 11,772 species (Speidel and Naumann 2004; Lafontaine and Fibiger 2006). Many species of owlet moths are considered an agricultural problem around the world. Their larvae are typically known as ‘cutworms’ or ‘armyworms’ due to enormous swarms that destroy crops, orchards and gardens every year. Plusiinae is a main large subfamily of Noctuidae, with more than 500 species worldwide, and they are spread from the tropics to the arctic (Ronkay et al. 2008). *Macdunnoughia hybrida* Ronkay, 1986, one of the species in Plusiinae, has been recorded in China, Korea, and Japan. *M. hybrida* is a polyphagous herbivorous moth, with the larvae feed on plants of Fabaceae and Cruciferae. Mitogenome can be utilized in research on taxonomic resolution and phylogeny, we sequenced the complete mitogenome of *M. hybrida* and analyzed the phylogenetic relationships of Noctuidae based on mitogenome data.

Six male adults of *M. hybrida* were collected from Xinyang City, Henan Province, China (32°03′N, 114°07′E, July 2019) and were stored in Entomological Museum of Anyang Institute of Technology (Accession number AIT-E-MH04, please contact Dr. Shuang Xue, email: xshk99@163.com). Total genomic DNA was extracted from muscle tissues of the thorax using DNeasy DNA Extraction kit (Qiagen, Hilden, Germany). A pair-end sequence library was constructed and sequenced using Illumina HiSeq 2500 platform (Illumina, San Diego, CA), with 150 bp pair-end sequencing method. A total of 32.4 million reads were generated and had been deposited in the NCBI Sequence Read Archive (SRA) with accession number SRR14250281. With the mitochondrial genome of *Diachrysia nadeja* (MT916722) employed as reference, raw reads were assembled using MITObim v 1.7 (Hahn et al. 2013). By comparison with the homologous sequences of other Noctuidae species from GenBank, the mitogenome of *M. hybrida* was annotated using software GENEIOUS R11 (Biomatters Ltd., Auckland, New Zealand).

The complete mitogenome of *M. hybrida* is 15,382 bp in length (GenBank accession no. MW924384), and contains the typical set of 13 protein-coding, two rRNA, and 22 tRNA genes, and one non-coding AT-rich region. Gene order was conserved and identical to most other previously sequenced Noctuidae (Timmermans et al. 2014; Li et al. 2015; Xue et al. 2019; Gao et al. 2021). The nucleotide composition of the mitogenome is 80.7% A + T content (A 38.9%, T 41.8%, C 11.4%, G 7.9%). Four PCGs (*nad4*, *nad4l*, *nad5* and *nad1*) were encoded by the minority strand (N-strand) while the other nine were located on the majority strand (J-strand). Except for *cox1* started with CGA, all other PCGs started with the standard ATN codons (seven ATG, four ATT and one ATA). Except for three genes (*cox1*, *cox2* and *nad4*) end with the incomplete stop codon T−, all other PCGs terminated with the stop codon TAA or TAG. The 22 tRNA genes vary from 63 bp (*trnR*) to 71 bp (*trnK*). Two rRNA genes (*rrnL* and *rrnS*) locate at *trnL1*/*trnV* and *trnV*/control region, respectively. The lengths of *rrnL* and *rrnS* in *M. hybrida* are 1,353 and 784 bp, respectively, with AT contents of 84.3% and 84.8%, respectively.

Phylogenetic analysis was performed based on the nucleotide sequences of 13 PCGs from 23 Noctuidae species. Alignments of individual genes were concatenated using SequenceMatrix 1.7.8 (Vaidya et al. 2011). Phylogenetic tree was constructed through raxmlGUI 1.5 (Silvestro and Michalak 2012). Phylogenetic analysis positioned *M. hybrida* in a well-supported clade with *Diachrysia nadeja* and three *Ctenoplusia* species (*C. agnate*, *C. albostriata* and *C. limbirena*) with high support value (Figure 1), indicating genus *Macdunnoughia* had a close relationship with *Diachrysia* and *Ctenoplusia* within Plusiinae. The relationships (Catocalinae + (Plusiinae + (Acronictinae + (Heliothinae + (Amphipyrinae + (Hadeninae + Noctuinae)))))) were supported within Noctuidae. The monophyly of Amphipyrinae and Noctuinae could not be confirmed by this phylogenetic tree. In conclusion, the mitogenome of *M. hybrida* is sequenced in this study and can provide essential DNA molecular data for further phylogenetic and evolutionary analysis of Noctuidae.

**Figure 1.**
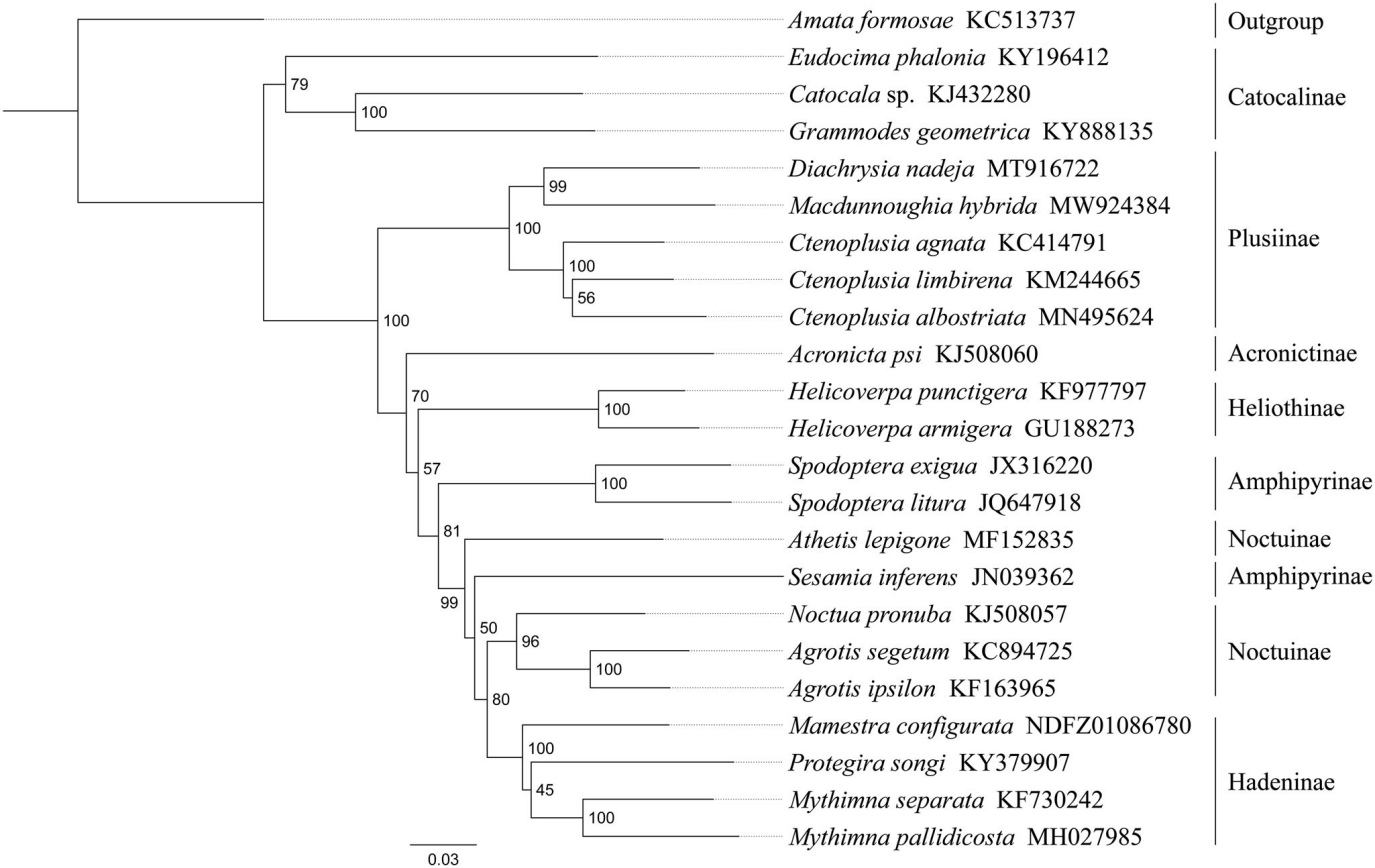
Phylogenetic relationships based on the 13 mitochondrial protein-coding genes sequences inferred from RaxML. Numbers on branches are Bootstrap support values (BS).

## Data Availability

The data that support the findings of this study are openly available in NCBI (National Center for Biotechnology Information) at https://www.ncbi.nlm.nih.gov/, reference number MW924384. The associated BioProject, SRA, and Bio-Sample numbers are PRJNA721792, SRR14250281, and SAMN18738784, respectively.
